# Ookinete-Specific Genes and 18S SSU rRNA Evidenced in *Plasmodium vivax* Selection and Adaptation by Sympatric Vectors

**DOI:** 10.3389/fgene.2019.01362

**Published:** 2020-02-21

**Authors:** Lilia González-Cerón, Mario H. Rodríguez, Marbella T. Ovilla-Muñoz, Frida Santillán-Valenzuela, Juan E. Hernández-Ávila, María Carmen Rodríguez, Jesús Martínez- Barnetche, Cuauhtémoc Villarreal-Treviño

**Affiliations:** ^1^ Regional Center of Research in Public Health, National Institute of Public Health, Ministry of Health, Tapachula, Mexico; ^2^ Vector Borne Diseases, Center for Research on Infectious Diseases, National Institute of Public Health, Ministry of Health, Cuernavaca, Mexico; ^3^ Chronic Infections and Cancer, Center for Research on Infectious Diseases, National Institute of Public Health, Ministry of Health, Cuernavaca, Mexico; ^4^ Center of Information for Public Health Decisions, National Institute of Public Health, Ministry of Health, Mexico City, Mexico

**Keywords:** *Plasmodium vivax*, *Nyssorhynchus albimanus*, *Anopheles pseudopunctipennis*, ookinete proteins, phylogenetic tree, 18S SSU rRNA, oocyst infection

## Abstract

In the southern Pacific coast of Chiapas, Mexico (SM), the two most abundant vector species, *Nyssorhynchus albimanus* and *Anopheles pseudopunctipennis*, were susceptible to different *Plasmodium vivax* Pvs25/28 haplotypes. To broaden our understanding of the existing *P. vivax* in the area, genes encoding proteins relevant for ookinete development and the 18S rRNA were studied. *P. vivax* infectivity (percentage of infected mosquitoes and oocyst numbers) was evaluated by simultaneously feeding infected blood samples from patients to *Ny. albimanus* and *An. pseudopunctipennis* female mosquitoes. Three infectivity patterns were identified: one group of parasites were more infective to *An. pseudopunctipennis* than to *Ny. albimanus*, another group was more infective to *Ny. albimanus*, while a third group infected both vectors similarly. In 29 parasite isolates, the molecular variations of ookinete-specific genes and the 18S rRNA-type S were analyzed. Using concatenated sequences, phylogenetic trees, and Structure analysis, parasite clustering within SM isolates and between these and those from other geographical origins were investigated. A ML phylogenetic tree resolved two parasite lineages: PvSM-A and PvSM-B. They were associated to a different 18S rRNA variant. PvSM-A parasites had 18S rRNA variant rV2 and correspond to parasites causing high oocyst infection in *Ny. albimanus*. A new ML tree and Structure analysis, both comprising global sequences, showed PvSM-A clustered with Latin American parasites. Meanwhile, all isolates of PvSM-B had 18S rRNA variant rV1 and remained as unique genetic cluster comprising two subgroups: PvSM-Ba, producing high infection in *An. pseudopunctipennis*, and PvSM-Bb, causing similar oocyst infection in both vector species. PvSM-A parasites were genetically similar to parasites from South America. Meanwhile, PvSM-B were exclusive to southern Mexico and share ancestry with Asian parasites. The results suggest that these lineages evolved separately, likely by geographic and vector restriction.

## Introduction

*Plasmodium vivax* causes more than 70% of malaria cases in America [Pan American Health Organization (PAHO), 2016]. At least 10 species of the family *Culicidae* transmit malaria in this continent. The Subgenera *Nyssorhynchus* and *Anopheles* diverged 100 million years ago, and *Nyssorhynchus* was recently raised to genus status (Foster et al., 2017; Harbach, 2018). *Nyssorhynchus albimanus and Anopheles pseudopunctipennis* are abundant in Mexico, Central America, and the northern South America regions (Sinka et al., 2012). For *P. vivax*, two 18S rRNA variants have been detected at different geographical sites (Prajapati et al., 2011); variant rV2, present in the Sal I reference strain, occurs in Central America. These parasites are highly infective to local *Ny. albimanus*; while Asian parasites expressing a different variant (rV1) produced low infections in this vector (Li et al., 2001).

In the Soconusco region of Chiapas, the southern part of Mexico that borders Central America, a *P. vivax* subpopulation was identified by a microsatellite variant c1 highly infective to *Ny. albimanus,* refractory to *An. pseudopunctipennis* (Joy et al., 2008), and similar to that of parasites with variant rV2 in Central America (Li et al., 2001). By contrast, subpopulations resolved by microsatellites f1/f2 were highly infective to *An. pseudopunctipennis,* and poorly infective to *Ny. albimanus* (Joy et al., 2008). The parasite variants' geographic distribution follows that of their susceptible vector and delineates populations structured according to their sympatric vector distribution.

Previously, we investigated in the same region the infectivity of *P. vivax* with ookinete surface proteins (Pvs25 and Pvs28) that participate directly in the parasite invasion of the mosquito midgut epithelium (Tomas et al., 2001). We documented that parasites Pvs25 87 Gln/130 Ile and Pvs28 87 Asn/110 Tyr were infective only to *Ny. albimanus*, while parasites Pvs25 with substitutions at 87 Gln-Lys/130 Thr and Pvs28 87 Asp/110 Asn were more infective to *An. pseudopunctipennis*. The analysis of a large number of isolates (n = 64) documented that these haplotypes predominate in the region, and no novel haplotypes were detected (Gonzalez-Ceron et al., 2010). These polymorphisms, described as *P. vivax* type-A (Sal I strain) and type-B, respectively, were apparently related to the parasite subpopulations identified by microsatellites in the region (Joy et al., 2008).

In this study, we broadened the molecular characterization of a sample of *P. vivax* parasites with different infectivity patterns to *Ny. albimanus* and *An. pseudopunctipennis* by analyzing genes expressed on the ookinete´s surface and micronemes as well as and ribosomal 18S SSU rRNA type-S variants. The genes included: Chitinase, involved in the parasite penetration of the mosquito midgut peritrophic matrix (Huber et al., 1991; Shahabuddin et al., 1993); CTRP (related to the circumsporozoite and thrombospondin adhesive protein), essential for ookinete motility and invasion of the midgut epithelium (Templeton et al., 2000; Limviroj et al., 2002; Li et al., 2004); CelTOS (cell-traversal protein of ookinetes and sporozoites), which participates in migration through the midgut epithelial cell cytoplasm (Kariu et al., 2006); and SOAP (secreted ookinete adhesive protein), involved in oocyst differentiation (Dessens et al., 2003).

Our results showed a polymorphism at CTRP and SOAP, and an association of the 18S rRNA variants to Pvs25 and Pvs28 variants, and to the PvSM-A and PvSM-B parasite lineages. They confirm the reported differential infectivity to the mosquito vectors and a possible adaptation process. A comparison of the ookinete-specific gene sequences to those reported in South America reveal an extended distribution of PvSM-A parasites, and the circumscription of PvSM-B to Southern Mexico.

## Materials and Methods

The protocol was approved by the Ethics Committee of the National Institute of Public Health, Mexico (CI1042). This study was carried out applying the bioethics guidelines (CITI program); all participants above 18 years old gave their written informed consent, and the minors between 7–17 years of age gave their assent accompanied by the written informed consent of one parent or guardian, in accordance with the Declaration of Helsinki.

### Study Site

The study was carried out in Chiapas, Mexico. Since 2000, the numbers of malaria cases in this region have fluctuated from 657 to 157 (< 1,000 each year) cases in this region and were produced by *P. vivax* only. Genetic studies had shown low rates of mixed genotype infections using microsatellites (Joy et al., 2008) or PvAMA-1 (Flores-Alanis et al., 2017). In this region, the two main malaria vectors are *Ny. albimanus,* highly prevalent in the rainy season, and *An. pseudopunctipennis*, highly prevalent in the dry season (Rodriguez et al., 2000).

### *Plasmodium vivax* Parasites and Mosquito Infections

Thick blood smears were examined from febrile patients seeking a malaria diagnosis at the Regional Center for Research on Public Health (CRISP, MNIPH) in Tapachula City, Chiapas, Mexico. Between 2001 and 2005, patients with at least 500 *P. vivax* parasites/µL of blood (7,000 white blood cells/µl was used as reference) were asked to donate five ml of venous blood. Infected blood samples were collected in heparin and centrifuged, and nonimmune human plasma was used to substitute the donor's plasma (Gonzalez-Ceron et al., 1999). To determine the infectivity of *P. vivax* in each blood sample, batches of female white-striped *Ny. albimanus* (A/WS-R) and *An. pseudopunctipennis* (P/TAP-R) from insectary colonies held at CRISP were simultaneously offered infected blood in artificial feeders (Gonzalez-Ceron et al., 2019). Only engorged mosquitoes were maintained under insectary conditions (Gonzalez-Ceron et al., 1999). Seven days post blood feeding, the proportion of infected mosquitoes and oocyst numbers in the midgut were recorded.

Ninety-three feeding experiments were carried out during the study period. Of these, 31 experiments resulted in no infected mosquitoes being observed. In 62 experiments, infection was detected in at least one mosquito species. In these experiments, the proportion of infected mosquitoes and the intensity of infection were investigated as well as the CSP genotype (in blood samples and the recovered salivary gland sporozoites). In 12 experiments, double infections were detected, according to the CSP genotype, and these were removed from further selection. From the remaining 50 infections, genetic analyses were conducted in all (seven) samples that were more infective to *Ny. albimanus* and all (six) samples with similar infectivity for *Ny. albimanus* and *An. pseudopunctipennis*, and (due to limited resources) we randomly selected 16 out of 37 samples more infective to *An. pseudopunctipennis*.

### Amplification and Sequencing of Ookinete Proteins Located at Different Chromosome

Whole DNA of each sample was extracted using the QIAamp DNA Blood Mini kit according to manufacturer's instructions (Qiagen, Valencia, CA, USA). Primers, PCR conditions, and amplification strategies are described below for each gene. PCR products with the correct molecular size were purified using the Wizard DNA Clean-Up System (Promega Madison WI, USA) or the MinElute PCR purification kits (Qiagen, Valencia, CA, USA) according to manufacturer's instructions unless otherwise indicated. Purified amplicons were Sanger sequenced at the High Throughput Genomics Unit (Department of Genome Sciences, University of Washington, Seattle, USA) or at Macrogen Inc. (Seoul, Republic of Korea).

#### Pvs25 and Pvs28 Proteins

For Pvs25, gene fragments comprising codons 87 and 130 were amplified with primers Pvs25-F23 and Pvs25-R214 (**Supplementary Table S1**). The amplification mixture contained 100 ng of total genomic DNA and the PCR mixture and conditions of amplification used were reported previously (Gonzalez-Ceron et al., 2019). For Pvs28, Pvs28-F and Pvs28-R primers flanking the coding region were used, and the PCR conditions used were reported previously (Gonzalez-Ceron et al., 2010).

#### Cell-Traversal Protein of Ookinetes and Sporozoites (CelTOS)

The complete gene located in Chr 14 was amplified using primers reported by Mehrizi (Mehrizi et al., 2017). PCR reactions were modified to use 100 ng of total DNA and 0.4 mM of each primer, 0.25 mM dNTPs, (Invitrogen, Carlsbad, CA,USA) 1X GoTaq Flexi buffer, 1.5 mM MgCl2 for 1.5 U of GoTaq Flexi DNA polymerase (Promega Madison WI, USA). Reaction conditions were as follows: 95°C for 3 min followed by 35 cycles of 95°C for 30 s, 57°C for 50 s, and 72°C for 60 s; these were held at 72°C for 5 min.

#### Secreted Ookinete Adhesive Protein (SOAP)

The *pvsoap* gene was amplified using primers pvSOAP-F and pvSOAP-R (**Supplementary Table S1**). For PCR reactions, 100 ng of total DNA, 0.25 mM of each primer, 0.25 mM dNTPs, 1X buffer (100 mM KCl, 20 mM Tris–HCl pH 7.5), and 2.4 mM MgSO_4_ for 2 U of High Fidelity PlatinumR Taq DNA polymerase (Invitrogen Carlsbad CA, USA) were used. Reaction conditions were as follows: 94°C for 2 min followed by 35 cycles of 94°C for 30 s, 60°C for 30 s, and 72°C for 45 s; these were held at 72°C for 5 min. DNA fragments were cloned using a kit (TOPO TA Cloning, Invitrogen Carlsbad CA, USA) according to manufacturer's instructions. Plasmids were extracted using the kit Wizard plus Minipreps DNA Purification System (Promega_Madison WI, USA).

#### Chitinase

First, differences in the chitinase gene sequence were investigated in two *P. vivax*-infected blood samples with different Pvs25/28 types: blood sample SM1804 had Pv25-Ile130 (type A) and blood sample SM0505 had Pv25 130Thr (type B) (Gonzalez-Ceron et al., 2010). pvChiti-F1, pvChiti-R1, pvChiti-F2, and pvChiti-R2 primers were used to amplify complete genes (**Supplementary Table S1**). For PCR reactions, 100 ng of DNA, 0.4 mM of each primer, 0.4 mM dNTPs, 1X buffer (100 mM KCl, 20 mM Tris–HCl pH 7.5), and 2 mM MgSO_4_ for 2.5 U of PlatinumR Taq DNA polymerase (PlatinumR Taq DNA polymerase High Fidelity, Invitrogen Carlsbad CA) were used. Reaction conditions were as follows: 94°C for 3 min followed by 35 cycles of 94°C for 30 s, 65°C for 30 s, and 68°C for 3 min; held at 72°C for 5min. Nucleotide sequences of the two samples were aligned using the corresponding sequence of the Sal I strain (XM_001613347; 1953 bp) as reference. As expected, the sequence from sample SM1804 was identical to that of Sal I, and SM0505 had two copies of a 54 bp fragment at the 5´end; therein, the complete chitinase gene sequences of the two isolates differed by 54 bp (1,953 vs 2007 bp) (**Supplementary Figure S1**). The identity of chitinase genes in the *P. vivax* samples was investigated according to the gene size identified above using pvRepChiti-F and pvRepChiti-R primers (**Supplementary Table S1**). For PCR reactions, 100 ng of total DNA and 0.2 mM of each primer, 0.2 mM dNTPs, 1X buffer (100 mM KCl, 20 mM Tris–HCl pH 7.5), and 2.4 mM MgSO_4_ for 2.5 U of PlatinumR Taq DNA polymerase (High Fidelity, Invitrogen Carlsbad CA, USA) were used. Reaction conditions were as follows: 95°C for 2 min followed by 35 cycles of 95°C for 45 s, 60°C for 45 s, and 72°C for 1 min; these were held at 72°C for 3 min. The approximate molecular size of the gene fragments was recorded (300 versus 354 bp).

#### Circumsporozoite and Thrombospondin-Related Adhesive Protein (CTRP)

The complete CTRP gene of ≈ 6Kbp (Kaneko et al., 2006) from isolate SM8101 (this produced high oocyst infection in *An. pseudopunctipennis*) was amplified. Primers flanking the coding region 4100-F and 4099-R were used (**Supplementary Table S1**). For PCR reactions,100 ng of DNA and 0.4 mM of each primer, 0.5 mM dNTPs, 1X buffer (100 mM KCl, 20 mM Tris-HCl, pH 7.5), and 2 mM MgSO_4_ for 2.5 U of PlatinumR Taq DNA polymerase (PlatinumR Taq DNA polymerase High Fidelity, Invitrogen Carlsbad CA, USA) were used. Reaction conditions were as follows: 94°C for 3 min followed by 35 cycles of 94°C for 1 min, 56°C for 45 s, and 68°C for 6 min; these were held at 68°C for 10 min. A fragment of 6,195 bp containing the CTRP ORF was amplified. The amplified product was cloned using pBluescript^®^ II SK (Stratagene Products La Jolla CA, USA) between the *Xho*I (Invitrogen Carlsbad CA, USA) and *Bam*HI (Invitrogen Carlsbad CA, USA) sites. To construct a CTRP library, 250 ng of cloned DNA was digested using 0.9 U of *Rsa*I (Invitrogen Carlsbad CA, USA) during 15 min at 37°C. The expected fragment lengths of the digested products ranged from 113–829 bp. Fragments cloned in the TOPO TA Cloning (Invitrogen Carlsbad CA, USA) were purified using the High Pure Plasmid Isolation kit (Roche Diagnostics Mannheim, Germany) following manufacturer's instructions.

The sequences were assembled using Sal I strain sequences as references: XM001614511.1 (Carlton et al., 2008) and AB47369.1 (Kaneko et al., 2006). To complete gaps, new PCR primers were designed: CT1-F, CT1-R; CT2-F, CT2-R; CT3-F, CT3-R, and CT4-F, CT4-R (**Supplementary Table S1**), which amplified four fragments of approximately 1,801, 1,701, 2,101, and 464 bp. For PCR reactions, 100 ng of DNA and 0.2 mM of each oligonucleotide, 0.5 mM dNTPs, 1X buffer (100 mM KCl, 20 mM Tris–HCl pH 7.5), and 2.4 mM MgSO_4_ for 2.5 U of PlatinumR Taq DNA polymerase (PlatinumR Taq DNA polymerase High Fidelity, Invitrogen Carlsbad CA, USA) were used. Reaction conditions were as follows: 95°C for 2 min, 35 cycles of 95°C for 45 s, 60°C for 45 s, and 72°C for 1.30 min; these were held at 72°C for 3 min. The four PCR fragments were amplified by triplicate.

Three gene fragments showing differential mutations in SM8101 compared to Sal I strain (**Supplementary Figure S1**) were analyzed for all parasites. A fragment of ≈ 450 bp containing a In/del segment (1564-2044F and R) was amplified with CT18rF and CT18rR primers. CT-2F and CT-2R primers amplified a fragment of ≈ 750 bp comprising codon 888. 1754-F and 1754-R primers were used to amplify a fragment of approximately of ≈ 350 bp that comprises codon 1754. In total, ≈ 1500 bp of the *ctrp* gene were examined in all parasite isolates. For PCR reactions, 100 ng of DNA and 0.2 mM of each primer, 0.5 mM dNTPs, 1X buffer (100 mM KCl, 20 mM Tris–HCl pH 7.5), and 2.4 mM MgSO_4_ for 2.5 U of PlatinumR Taq DNA polymerase (PlatinumR Taq DNA polymerase High Fidelity, Invitrogen Carlsbad CA, USA) were used. Reaction conditions were as follows: 95°C for 3 min followed by 35 cycles of 95°C for 45 s, 60°C for 45 s, and 68°C for 1 min; these were held at 72°C for 3 min.

### 18s rRNA Type-S

To investigate size variations of the ribosomal fragment, gene amplifications were carried out using SSU-F and SSU-R primers (Prajapati et al., 2011). For PCR reactions, 0.4 mM of each primer, 0.4 mM dNTPs, 1X buffer (100 mM KCl, 20 mM Tris–HCl pH 7.5), 2.4 mM MgSO_4_ for 2 U of Platinum Taq DNA polymerase (Invitrogen Carlsbad CA, USA), and 100 ng of DNA were used. The reaction conditions were as follows: 95°C for 5 min, followed by 35 cycles of 95°C for 45 s, 55°C for 45 s, and 72°C for 45 s; these were held at 72°C for 5 min. The two rV1 (480 bp) and rV2 (450 bp) variants were distinguishable. To confirm the identity of these variants, DNA sequences from samples of different molecular sizes, cloned in TOPO TA Cloning (Invitrogen Carlsbad CA; USA), were obtained.

### Gene Sequences

The quality of pherograms of the nucleotide sequences were examined using BioEdit v7 (Hall, 1999) and by manual examination. The nucleotide sequences from different genes were aligned using CLUSTALW in BioEdit, using reference sequences as follows: Pvs25 (XM_001608410.1) (Carlton et al., 2008), Pvs28 (XM_001608411.1) (Carlton et al., 2008), CTRP (XM001614511.1 (Carlton et al., 2008), AB47369.1 (Kaneko et al., 2006) and PVP01_0824900 (http://plasmodb.org), SOAP (XM_001616857) (Carlton et al., 2008), CelTOS (AB194053.1) (Kariu et al., 2006), and Chitinase (XM_001613347) (Carlton et al., 2008). For the 18S SSU rRNA S-type, the sequences of the two variants were used: type Sal I (rV2) (U03080.1 and AAKM01000006.1) (Li et al., 2001; Prajapati et al., 2011) and type Thai or variant rV1 (U93234.1) (Li et al., 1997). The following sequences were used in the analysis: Pvs25 (EU024410.1, EU024411.1, EU024414.1, EU024416.1, EU024417.1, EU024419.1, EU024420.1, EU024436.1-EU024438.1, EU024449, EU024455.1, EU024458.1, EU024466.1- EU024472.1), and Pvs28 sequences (EU514769.1-EU514785.1, E U514788.1, EU514789.1) (Gonzalez-Ceron et al., 2010). Gene sequences obtained from this study were submitted to NBCI GenBank [accession numbers: Pvs25 (MN15360-9), Pvs28 (MN15370-8), SOAP (MK992255-84), CTRP (MK992285-2369), Chitinase (MN15379-80), and CelTOS (MN15381-409)].

### Genetic Analysis

Multi-gene haplotypes for southern Mexican isolates were resolved using all single nucleotide variations (SNV) and In/dels detected at different locus. To analyze the linkage equilibrium of each multi-locus haplotype, we built a matrix with all haplotypes (rows) and loci (columns) described in **Supplementary Table S2** as input for the LIAN V 3.7 software (Haubold and Hudson, 2000). In linkage equilibrium, alleles at each *loci* were assorted independently. LIAN simulates random allele assortment at each *loci* and tests the difference between observed (*V*_D_) and the expected variance (*V*_e_), where *H*_0_: *V*_D_ = *V*_e_. The statistical difference between *V*_D_ and *V*_e_ (i.e., linkage disequilibrium (LD)) was assessed by computing a *P-*value by Monte Carlo (PMC) simulations with 10,000 re-samplings without replacement.

For the genetic analyses working under the assumption that SNVs are more informative and might have similar mutation rates, In/dels of faster mutation rates were excluded. To assign genetic clusters for parasites studied in here, gene sequences of the ookinete proteins were concatenated (SM) and used to construct one Maximal likelihood phylogenetic tree, as were bootstrapping with 1,000 replications and the substitution model HKY+G in MEGA v6.0 (Tamura et al., 2013).

To search for genetic similarities between southern Mexican parasites and those from South America, homologous gene sequences of *P. vivax* parasites from the PlasmoDB database (http://plasmodb.org/plasmo/) were obtained (**Supplementary Table S3**). Ookinete-specific gene sequences from this study and those obtained from PlasmoDB were trimmed to generate a new sequence (concatenated-G).

Using concatenated-G, a new Maximal likelihood phylogenetic tree was constructed as indicated above. The population structure in the global sample was estimated with k ranging from 2 to 6 and 20 replicates. Markov chain Monte Carlo (MCMC) examination used 50,000 “burn in” steps followed by 100,000 iterations under the add mixture model using the clustering algorithm STRUCTURE v2.3.4 (Pritchard et al., 2000). To define the most probable K, the probabilities of LNP [D/K] and Delta K were calculated using the Evanno test, Web version: v0.6.94 (July 2014) (Earl and vonHoldt, 2012). To determine the degree of differentiation within and between *P. vivax* clusters in southern Mexico, and with those from other geographic sites, Wright's fixation statistics (F*_ST_*) (Wright, 1951) values were calculated using the model of two parameters of Kimura 2P and statistics output (Chi-square at 95% CI) in DnaSP v6 (Rozas et al., 2017). F*_ST_* values range from 0 to 1; high values indicate high degree of differentiation between the two parasite populations. DNA polymorphisms and genetic parameters were analyzed for each parasite lineage of southern Mexico and parasites grouped by their geographical origin. The number of segregating sites (S), mutations or nucleotide changes (M), haplotypes (H), haplotype diversity (Hd), and nucleotide and genetic diversity (π) were calculated with DnaSP v5.1 software (Rozas et al., 2017). An unpaired t test was carried out at the 95% confidence using Stata.

### Statistical Analysis

Differences in the proportion of infected mosquitoes were analyzed using logistic regression assuming a binomial distribution, and the intensity of infection (number of oocyst per infected mosquito) was compared with a negative binomial model (McCullagh and Nelder, 1989). The statistical significance of the differences, for both the proportion of infected mosquitoes and intensity of infection, was estimated by the linear combination of the regression coefficients. We summed up the mosquito numbers in each genetics group to have a sample size large enough to carry out the statistical modeling, but we calculated the variance–covariance matrix of the coefficients and their linear combinations using clustered robust estimator (Royall, 1986; Rogers, 1993). This methodology allowed for the modeling of clustered data from different experiments, taking into consideration the correlation within each parasite sample (experiment), but used the pooled data in the model. Pooling data on different experiments (parasite samples) is a common statistical approach in the analysis of differential infection susceptibility (Gonzalez-Ceron et al., 1999; Rodriguez et al., 2000; Gonzalez-Ceron et al., 2007; Gonzalez-Ceron et al., 2010; Gonzalez-Ceron et al., 2019). **Supplementary Table S2** shows the proportion of infected mosquitoes and the intensity of infection in each parasite sample. All significance tests were carried out at the 95% confidence level using the Wald test; this statistical test is a classical method for hypothesis testing of the coefficients of a regression model and is based on the weighted distance (the standard error of the coefficient) of the estimated value of coefficient and a hypothesized value (Altman, 1991). All significance tests were carried out at the 95% confidence level using Stata 14^©^ Stata Corporation.

## Results

### Molecular Polymorphisms

#### Gene Polymorphism of Ookinete Proteins

No signs of mixed genotype infections were detected in any of the gene fragments. Non-synonymous polymorphisms (Glu87Lys and Ile130Thr or Asn87Asp and Tyr110Asn), at *pvs*25 (187–540 nt; 354 bp) or *pvs28* (88–507 nt; 420 bp), respectively were detected in 22 isolates. The other seven isolates had a similar sequence to that of the Sal I strain. A fragment of 369 bp (82–450 nt) from the *soap* gene was obtained. In 29 parasites, 23 had a non-synonymous substitution at codon Met74Val. Three of these had a synonymous substitution at codon Asn35, and another six were similar to the Sal I strain (**Table 1**).

**Table 1:** Genetic mutations and amino acid polymorphism of ookinete proteins and 18S rRNA type-S variant in 29 isolates from southern Mexico.

# of haplotype	N	18S rRNA type-S:	Ookinete genes: codons numbers and nucleotides, and amino acid change																	
			**Pvs25**		**Pvs28** ^1^			**SOAP**		**CTRP**					**CelTOS**				**Chitinase^3^**
			**87**	**130**	**87**	**110**	**repeats**	**35**	**74**	**repeat type^2^**	**888**	**1012**	**1754**	**1755**	**37**	**118**	**178**	**179**	**Chit1/Chit2**
			**Sal I strain:**																	
		**rV2**	**Q/cag**	**I/atc**	**N/aat**	**T/tat**	**R6**	**N/aac**	**M/atg**	**R1**	**M/atg**	**Q/gaa**	**G/ggt**	**R/cgt**	**R/cgg**	**V/gtg**	**K/aag**	**G/ggt**	**Chit1**
1	1	.	.	.	.	.	.	.	.	R2	.	.	.	.	agg	.	T/acg	.	.
2	1	.	.	.	.	.	.	.	.	R2	.	.	.	.	agg	.	.	.	.
3	1	.	.	.	.	.	.	.	.	R3	.	.	.	.	agg	.	.	.	.
4	1	.	.	.	.	.	.	aat	.	R1	.	.	.	.	agg	.	.	.	.
5	1	.	.	.	.	.	.	aat	.	R3	.	.	.	.	agg	.	.	.	.
6	1	.	.	.	.	.	.	aat	.	R4	.	gag	.	.	agg	L/ctg	T/acg	R/cgt	.
7	1	.	.	.	.	.	.	.	V/gtg	R4	.	gag	.	S/agt	agg	.	.	.	.
8	8	rV1	.	T/acc	D/gat	N/aat	R5^1^	.	V/gtg	R2	K/aag	.	C/tgt	.	agg	.	.	.	.
9	3	rV1	.	T/acc	D/gat	N/aat	R5	.	V/gtg	R2	K/aag	.	C/tgt	.	agg	.	.	.	Chit2
10	1	rV1	.	T/acc	D/gat	N/aat	R5	.	V/gtg	R2	K/aag	.	C/tgt	.	.	.	.	.	Chit2
11	4	rV1	K/aag	T/acc	D/gat	N/aat	R5	.	V/gtg	R2	K/aag	.	C/tgt	.	agg	.	.	.	.
12	5	rV1	K/aag	T/acc	D/gat	N/aat	R5	.	V/gtg	R2	K/aag	.	C/tgt	.	agg	.	.	.	Chit2
13	1	rV1	K/aag	T/acc	D/gat	N/aat	R5	.	V/gtg	R2	K/aag	.	C/tgt	.	.	.	.	.	Chit2

1 A deletion of one repeat amino acid sequence GSGGE. ^2^repeat variation at the amino end of CTRP (nt 1580–2222). ^3^Chit1: Sal I type had one copy of domain NGSGSGSGGETETGGESG, and chit2 had two copies of that domain. Gene fragments analyzed: pvs25, nt 187–540 (354 bp); pvs28, complete gene; soap, nt 70–450 (381 bp); celtos, complete gene (588 bp); ctrp, nt 1580–2022 (450 bp); nt 2305–3114 (810 bp); nt 5077–5434 (357 bp). Dots indicate no variation compared to the reference strain. Single-letter amino codes are shown. nt, nucleotide.

The complete *celtos* gene was obtained. Twenty-seven isolates had a synonymous change at codon Arg37. Three non-synonymous changes were detected; two isolates had a change at codon Lys178Thr, one of them had two additional changes at codons Val118Leu and Gly179Arg. Other isolates were similar to the Sal I strain (**Table 1**).

The complete gene sequence (6,360 bp) of *ctrp* was obtained from isolate SM8101, and a comparison to the Sal I sequence (XM001614511.1) showed several mutations (**Supplementary Figure S1**). At the von Willebrand type A (vWA) domain, a variation due to insertions and deletions (In/dels) between the second and third vWA domains, one non-synonymous mutation at codon 888Met (atg) → Lys (aag), and one synonymous mutation at codon 1283 Pro (cct→ccc) were documented. In addition, an insertion of 36bp at the transmembrane domain (TM) was detected.

In the thrombospondin type-1 (TPS1) domains, the gene fragment nt 5208–5443 reported by Kaneko et al. (AB247369.1) (Kaneko et al., 2006) and detected in our isolates was absent in the Sal I sequence reported by Carlton (XM001614511.1) (Carlton et al., 2008). In this fragment, the SM8101 had a non-synonymous polymorphism at codon 1754Gly (ggt) → Cys (tgt). All gene variations were consensed by 3x-5x using forward and reverse primers.

The analysis of DNA fragments comprising In/dels and codons 888 and 1754 in CTRP for all parasites showed four different In/del patterns within nucleotides 1580–2022 (**Table 1**; **Supplementary Figure S1**). Nucleotide changes in gene fragments of 810 bp (nt 2305–3114) and 357 bp (nt 5077–5434), Met888Lys and Gly1754Cys, were detected in 22 of the 29 isolates. Two isolates lacking these mutations (SM15202 and SM1804) had a synonymous change at codon 1012Glu, and the isolate SM1804 had an additional non-synonymous change at codon Arg1755Ser. The gene polymorphisms (SNV and In/dels) detected at genes expressed in ookinetes resolved 13 haplotypes (**Table 1**).

The *chitinase* gene from isolate SM1804 was identical to that of Sal I, and isolate SM0505 had two copies of the domain NGSGSGSGGETETGGESG (inserted between codons 99 and 100). Size variation analysis of a fragment containing this domain revealed that 19 and 10 parasite isolates had a short fragment (Sal I-like of 300 bp) and a larger fragment (resembling 354 bp), respectively (**Table 1**; **Supplementary Figure S2**).

#### Determination of 18S rRNA Type-S

Seven of the 29 isolates had the rRNA 18SSU S rV2 variant and the others had the rV1 variant. The molecular size of these isolates matched the respective DNA sequences (**Table 1**; **Supplementary Figure S3**).

### Genetic Analysis

Considering SNV and In/dels from altogether genes fragments, 13 haplotypes were resolved (**Table 1** and **Supplementary Table S3**). The multi-locus LD analysis of all haplotypes (rows) and 18 loci (columns) showed an overall observed variance (*V*_D_) of 21.83 *versus* an expected variance (*V*_e_) of 3.91. The difference between *V*_D_ and *V*_e_ was significant as revealed by Monte Carlo simulation (*H*_0_: *V*_D_ = *V*_e_, *P* < 1.0 x 10^-4^). This indicates that the observed haplotype structure differs from the expected from random mating and a strong LD between the tested *loci*. The genetic diversity of each locus in shown in **Supplementary Table S3**.

#### Two Genetic Lineages of *Plasmodium vivax* Were Identified in Southern Mexico

**Figure 1:**
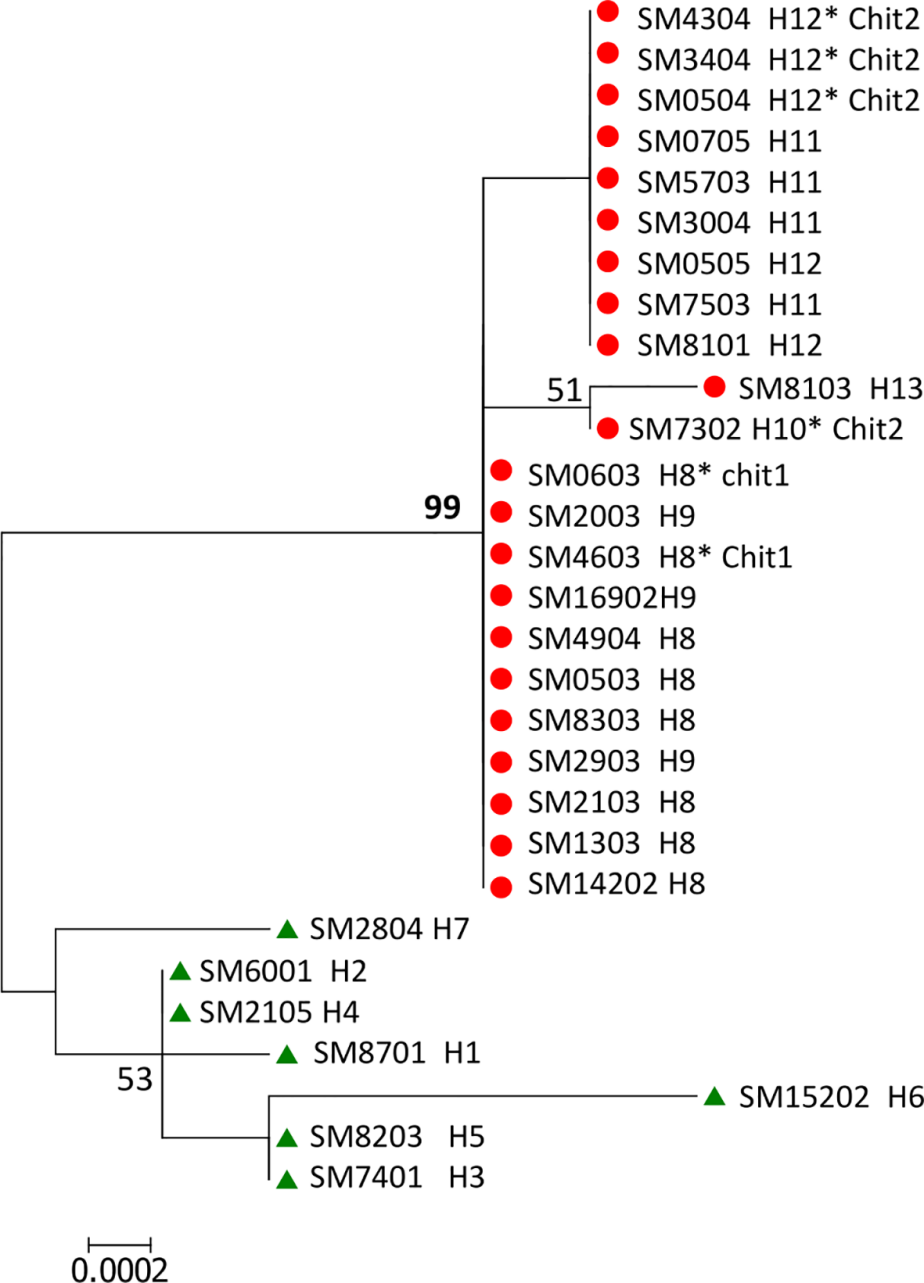
ML phylogenetic tree of *Plasmodium vivax* gene sequences of ookinete proteins defined two parasite lineages in southern Mexico. A concatenated nucleotide sequence of 2910 pb from 29 isolates was used to build a maximum likelihood tree with 1000 bootstraps using a HKY+G substitution model in MEGA v6.0. The numbers shown along nodes indicate bootstrap values. The concatenated sequence comprises *pvs25*, *pvs28*, *soap*, *celtos*, and *ctrp* gene fragments. The topology shows two genetic lineages named PvSM-A (in green) and PvSM-B (in red) with 99% bootstraps. Isolate codes and haplotype (H) numbers are indicated. PvSM-B lineage comprised two phenotypes; PvSM-Ba, highly infectious to *An. pseudopunctipennis,* and those caused similar infection in both vector species, designated as PvSM-Bb*.

Gene polymorphisms at codons Pvs25 130, Pvs28 87 and 110, SOAP 74, and CTRP 888 and 1754 correlated to the variant of ribosomal 18S type S. Accordingly, the ML phylogenetic tree with the 29 (SM) concatenated sequences of 2910 bp supported two genetic clusters by a 99% bootstrap value and correspond to lineages PvSM-A and PvSM-B to indicate their southern Mexican origin (**Figure 1**). The PvSM-A lineage was comprised by the seven parasite samples more infective to *Ny. albimanus* and seven haplotypes (H1-H7) and had 18S rRNA variant rV2. Meanwhile, PvSM-B lineage [which includes parasites highly infective to *An. pseudopunctipennis* (PvSM-Ba) and those equally infective for both mosquito species (PvSM-Bb)] was comprised of 22 samples and six haplotypes (H8-H13) and had 18S rRNA variant rV1 (**Table 1**).

#### Genetic Similarities and Structure of *Plasmodium vivax* Ookinete Protein Within Parasite Lineages From Southern Mexico (SM) and Between Them and Those From Other Geographical Sites

The global concatenated sequence of 2478 bp did not comprise a CTRP gene fragment containing codon 1754 because CTRP sequences from PlasmoDB lacked information at this position (**Supplementary Figure S1**). The differences between concatenated sequences -SM and -G are indicated in **Supplementary Table S4**. The southern Mexican parasites from this study and those from PlasmoDB (using concatenated-G) showed similar clustering as shown in **Figure 1**, and only one new haplotype was discovered in PvSM-A lineage (see **Supplementary Figure S4**).

The comparison of polymorphisms between southern Mexican parasites and to those of different geographical origin revealed that the mutation at the CTRP codon 888 was exclusive of lineage PvSM-B (**Supplementary Table S5**). Mutations at Pvs25 codon 130 and Pvs28 codons 87 and 110, simultaneously present in lineage PvSM-B, were also present in all parasites from outside the American continent. The single mutation at Pvs28 codon 110 was observed in parasites from Peru. Polymorphisms at SOAP were only detected in Latin American parasites; a mutation at codon 35 was present in nine parasites from Colombia and six parasites from southern Mexico (lineage PvSM-A), whereas, a mutation at codon 74 was present in lineage PvSM-B, one parasite of lineage PvSM-A, and three Peruvian parasites. In CelTOS, mutations at codons 178 and 179 were detected in parasites from SM. Yet only the mutation at codon 178 was present in parasites from outside the American continent, while the mutation at codon 118 was present in parasites from different geographical origins. In addition, only one haplotype of the PvSM-A lineage had the three mutations (codons 118, 178 and 179) (**Supplementary Table S5**).

The concatenated multi-gene sequence consisting of 109 global parasites (including Sal I and Belem strains) and 2478 bp had four synonymous and 36 non-synonymous mutations, and 53 haplotypes (**Supplementary Table S5**). Parasites of lineage PvSM-A from southern Mexico had similar nucleotide diversity to parasites from Colombia and Peru (t-statistic -8.25, df = 56, *P < 0.0001* and t-statistics -6.10, df = 61, *P < 0.000*, respectively), while lineage PvSM-B showed the lowest nucleotide diversity. The Latin American parasites had lower nucleotide diversity than those from outside this continent (t-statistic 44.61, df = 107, *P = 0.0001*). As expected, the nucleotide diversity of all 39 parasites from southern Mexico (29 from this study and 10 from PlasmoDB) was intermediate to that calculated for PvSM-A and PvSM-B (**Table 2**).

**Table 2:** Comparison of parameters of genetic diversity of *P. vivax* ookinete-specific genes between southern Mexican lineages and parasites from other geographical origins.

Geographic origin	N	Segregating sites	Mutations	synonymous changes	Non-synonymous changes	# Haplotypes	Haplotype diversity ± SD	Nucleotide diversity (Pi) ± SD
Lineage PvSM-A	7	6	6	2	4	5	0.905 ± 0.103	0.00096 ± 0.00027
Lineage PvSM-B	22	2	2	1	1	4	0.606 ± 0.062	0.00028 ± 0.00004
^1^southern Mexico	39	12	12	3	9	10	0.765 ± 0.04	0.00121 ± 0.00021
Peru	24	8	8	2	6	11	0.92 ± 0.029	0.00093 ± 0.0001
Colombia	19	6	7	2	5	12	0.947 ± 0.03	0.0008 ± 0.00007
^2^Latin America	84	16	17	3	14	29	0.929 ± 0.014	0.00162 ± 0.00006
Outside America	25	27	29	2	27	24	0.997 ± 0.012	0.00252 ± 0.00015

1 All sequences from this study (29) and from PlasmoDB (10). ^2^Includes all sequences from Latin America. Concatenated gene sequence of 2,478 bp [ctrp 771 bp (nt 2305–3077); pvs25 354 bp (nt 772–1125); pvs28 420 bp (nt 1126–1545), soap 381 bp (nt 1546–1926); and celtos 552 bp (nt 1927–2478)]. Sal I sequence (XM_001614511, XM_001608410, XM_001608411, XM_001616857, XM_001617213; Carlton et al., 2008).

**Figure 2:**
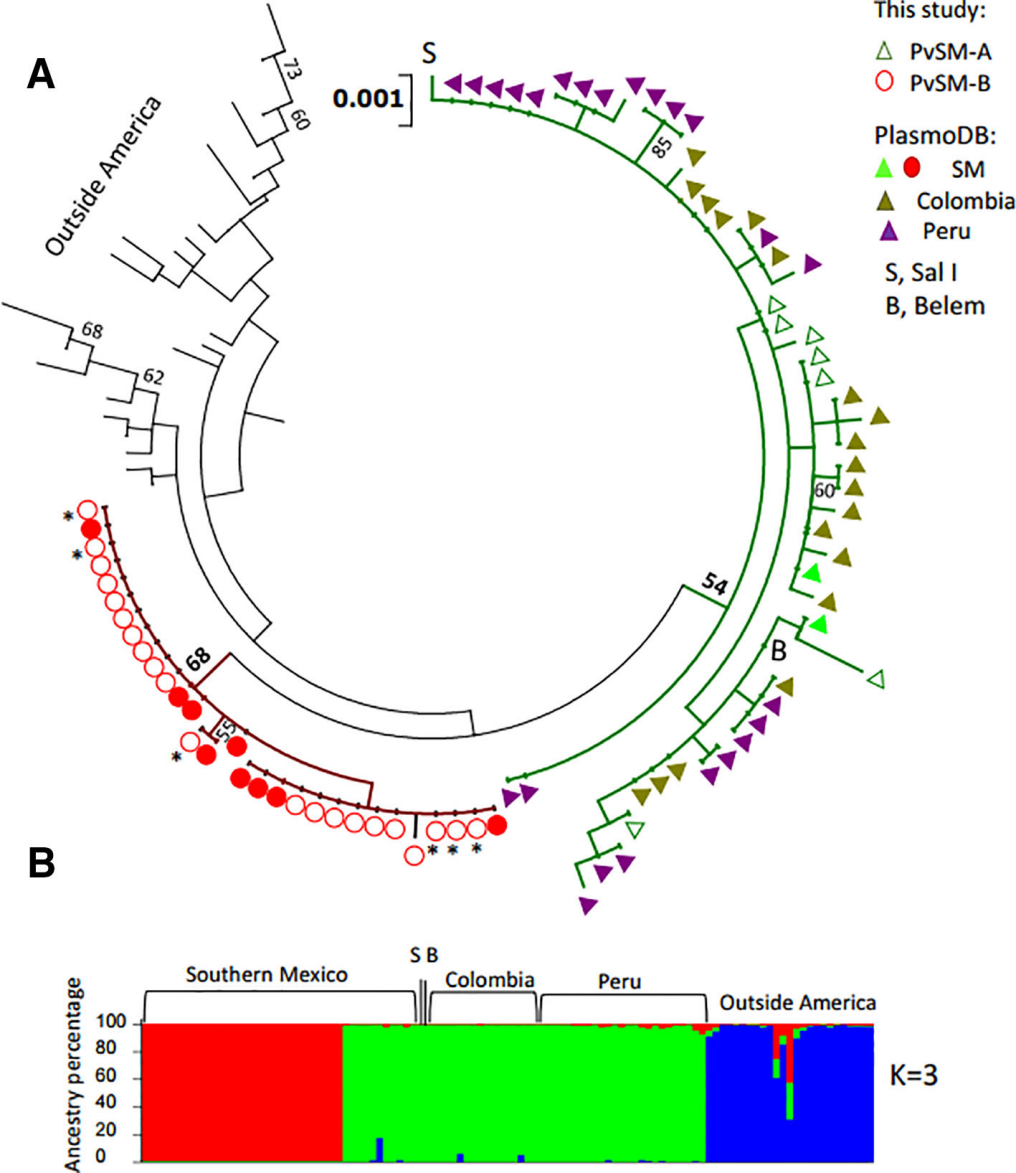
Phylogenetic tree and genetic structure of concatenated gene sequences of *Plasmodium vivax* ookinete proteins of southern Mexican and worldwide isolates. **(A)** Maximum Likelihood method based on the HKY+G model. The phylogenetic reconstruction tree was run with 1,000 replicates. Isolates of PvSM-A lineage and others from America clustered together (green branch), while another batch of isolates from southern Mexico clustered separately, including PvSM-B lineage (red branch). Parasites from outside Latin America are comprised in black branches. Bootstrap values above 50% are indicated; *indicates PvSM-Bb parasites. **(B)** Genetic structure analysis resolved three subpopulations as the most probable, each indicated by different colors: red shows only SM isolates, green shows PvSM-A plus other parasites from Latin America and blue shows parasites from outside America that clustered in one population. S, Sal I strain; B, Belem strain. The analysis involved 109 global nucleotide sequences of 2478 bp. *Pvs25*, *pvs28*, *soap celtos*, and *ctrp* gene fragments were included.

The phylogenetic tree showed that parasites from Peru and Colombia clustered within the PvSM-A lineage, whereas parasites from the PvSM-B lineage remained as a private cluster. Furthermore, parasites from outside Latin America formed a separate cluster (**Figure 2A**). The genetic structure analysis clustered parasite isolates into three subpopulations (**Figure 2B**) and correlated with the clustering resolved by the ML tree (**Figure 2** and **Supplementary Figure S5**). PvSM-B parasites from southern Mexico shared genetic ancestry with Madagascar parasites.

In accordance with those results, highly differentiated values were observed between lineages PvSM-A and PvSM-B (F*_ST_* = 0.7702). There were also high F*_ST_* values between PvSM-B lineage and parasites from Colombia (Chi = 40, df = 15, *P < 0.001*), Peru (Chi = 45, df = 14, *P* < 0.001), and from outside Latin America (*P* = 0.0127) (**Table 3**). The lowest F*_ST_* values occurred between lineage PvSM-A and parasites from Colombia and Peru, but they were not significant (**Table 3**). Additionally, a high fixation index (F*_ST_* = 0.3796) between parasites from Latin America and those from outside America was recorded (Chi2 = 109, df = 52, *P < 0.0001*). However, no genetic differences were observed between groups PvSM-Ba and PvSM-Bb (F*_ST_* = -0.117; *P = NS*). The differentiation value by comparing PvSM-A vs PvSM-Ba (F*_ST_* = 0.787; *P = 0.0017*) or PvSM-Bb (F*_ST_* = 0.773; *P = 0.072*) was similar.

**Table 3:** F*_ST_* values within *P. vivax* PvSM lineages from southern Mexico and between them and parasites from other geographical origins based on gene sequences of ookinete proteins.

	PvSM-A	PvSM-B	Peru	Colombia
PvSM-B	0.7702***	-		
Peru	0.1292	0.7457***	-	
Colombia	0.0110	0.8021***	0.1304	-
Outside America	0.5118	0.5444*	0.5093*	0.5372

Concatenated sequence comprised 2,478 bp: ctrp 771 bp (nt 2305–3077); pvs25 354 bp (nt 772–1125); pvs28 420 bp (nt 1126–1545), soap 381 bp (nt 1546–1926); and celtos 552 bp (nt 1927–2478). Overall chi-square = 316.111, P-value = 0.0000 ***; (df = 200). *, 0.01 < P < 0.05; **, 0.001 < P < 0.01; ***, P < 0.001.

### *Parasite* Lineages and Vector Infection

#### *Plasmodium vivax* Genetic Lineages and Mosquito Infectivity in Southern Mexico

The primary analysis of the proportion and intensity of infection suggested three different groups, one infecting mainly *Ny. albimanius* (PvSM-A); a second group, PvSM-Ba, infecting mainly *An. pseudopunctipennis*; and the third group, PvSM-Bb, infecting similarly both mosquito species (**Supplementary Table S3**). Our models confirmed that, when both species of mosquitoes were fed with parasites of lineage PvSM-A (7 experiments), the probability of infection of *Ny. albimanus* was 81 times higher (95% CI 14.98–433.53) than that of *An. pseudopunctipennis* (logistic model Wald Test, z *=* 5.11, df = 365, *P < 0.0001*). The oocyst infection intensity was 7.66 times greater (95% CI 3.79–15.49) in infected *Ny. albimanus* than in infected *An. pseudopunctipennis* (z = 5.66, df = 127, *P < 0.0001*).

In experiments using PvSM-Ba, our models confirmed that the proportion of infection was 31 times higher in *An. pseudopunctipennis* (95% CI 14.65–66.90) compared to *Ny. albimanus* (z = 8.89, df = 985, *P < 0.0001*), and the intensity of oocyst infection in *An. pseudopunctipennis* was 2.3 (CI 1.50–3.67) times higher than in *Ny. albimanus* (z = 3.53, df = 268, *P < 0.0001*) (**Table 4**). For the six experiments grouped into PvSM-Bb, our model corroborated that there were no statistical differences between both vector species in the proportion of infection (95% CI = 0.55–1.86; z = 0.03, df = 269, *P = 0.98*) and in the oocyst intensity (95% CI = 0.60–1.27; z = -0.71, df = 118, *P = 0.48*).

**Table 4:** *P. vivax* lineages in southern Mexico: oocyst infection rate and intensity by mosquito species.

*P. vivax* Genetic group	N experiments	Percentage of infected Mosquitoes (N)		Intensity of infection^3^; median of oocyst per mosquito (IQR)	
		*Ny. albimanus*	*An. pseudopunctipennis*	*Ny. albimanus*	*An. pseudopunctipennis*
PvSM-A	7	68.9% N = 180	2.7% N = 187	21 (6.5–72)	2 (1–9)
PvSM-B	22	11.6% N = 749	59.5% N = 509	8 (2–20)	25 (8–45)
^1^Subgroup PvSM-Ba	16	6.0% N = 638	66.5% N = 349	5 (1–15)	30.5 (8.5–53)
^2^Subgroup PvSM-Bb	6	44.1% N = 111	44.4% N = 160	11 (4–26)	16 (8–25)

1 Experiments with higher oocyst infection rate in An. pseudopuntipennis than in Ny. albimanus and ^2^experiments with no statistical difference in the oocyst infection rate in both vector species. IQR, interquartile range. ^3^Only infected mosquitoes are included in the calculation of the intensity of the infection.

The comparison of the mosquito infections produced by different parasite lineages or subgroups revealed that PvSM-A parasites produced a higher infection rate and oocyst intensity in *Ny. albimanus* than parasites PvSM-B and its subgroups PvSM-Ba or PvSM-Bb (**Table 5**). PvSM-B parasites and subgroups PvSM-Ba or PvSM-Bb produced a higher infection rate and oocyst intensity in *An. pseudopunctipennis* than PvSM-A parasites. PvSM-Bb parasites produced higher infection rates compared to PvSM-Ba in *Ny. albimanus*, but there was no difference in oocyst intensity. However, PvSM-Ba produced a higher infection rate and oocyst density in *An. pseudopunctipennis* than PvSM-Bb (**Table 5**).

**Table 5:** Statistics of the comparison of mosquito species infection rate and oocyst intensity between *P. vivax* lineages and subgroups.

*P. vivax* lineages and subgroups	Times higher in the prop. of infected mosquitoes (CI)	Statistics	*Times higher in the oocyst intensity (CI)	Statistics
*Ny. albimanus*				
PvSM-A vs PvSM-B	16.85 (5.27–53.8)	z = 4.77, df = 927, *P < 0.0001*	2.37 (1.08–5.20)	z = 2.16, df = 209, *P < 0.031*
PvSM-A vs PvSM-Ba	35.0 (10.44–116.72)	z = 5.78, df = 816, *P < 0.0001*	2.83 (1.26–6.36)	z = 4.52, df = 160, *P < 0.012*
PvSM-A vs PvSM-Bb	2.8 (0.86–9.13)	z = 1.71, df = 289, *P < 0.087*	2.1 (0.84–5.22)	z = 1.60, df = 171, *P < 0.109*
PvSM-Bb vs PvSM-Ba	12.47 (4.22–36.88)	z = 4.57, df = 747, *P < 0.0001*	1.35 (0.63–2.84)	z = 0.78, df = 87, *P = 0.437*
*An. pseudopunctipennis*				
PvSM-B vs PvSM-A	53.5 (10.36–276.65)	z = 4.75, df = 694, *P < 0.0001*	5.52 (4.27–7.13)	z = 13.04, df = 306, *P < 0.0001*
PvSM-Ba vs PvSM-A	72.18 (13.87–357.72)	z = 5.08, df = 534, *P < 0.0001*	6.24 (4.81–8.07)	z = 13.90, df = 235, *P < 0.0001*
PvSM-Bb vs PvSM-A	29.04 (4.69–179.90)	z = 7.01, df = 345, *P < 0.0001*	3.18 (2.26–4.49)	z = 6.61, df = 74, *P < 0.0001*
PvSM-Ba vs PvSM-Bb	2.49 (0.99–6.19)	z = 1.95, df = 507, *P < 0.059*	1.96 (1.29–2.96)	z = 3.18, df = 301, *P = .001*

CI at 95%; df, degree of freedom; CI, confidence interval. *Only considering infected mosquitoes.

## Discussion

Based on genes located at different chromosomes that code for surface and microneme proteins from the ookinete stage along with ribosomal rRNA variants, our findings highlight the presence of two *P. vivax* lineages in southern Mexico. These lineages differed in their infectivity pattern to *Ny. albimanus* and *An. pseudopunctipennis*. PvSM-A lineage, highly infective to *Ny. albimanus*, clustered with South American parasites, while PvM-B lineage comprising closely related parasites (PvSM-Ba and PvSM-Bb) seems to be exclusive to southern Mexico. PvSM-Bb parasites caused higher oocyst infection rates in *An. pseudopunctipennis* that to *Ny. albimanus*. PvM-Bb isolates, meanwhile, revealed adaptive tradeoffs to both vector species without expressing genetic differences from PvSM-Ba.

Previously, based on microsatellite analysis, three *P. vivax* subpopulations were identified in our study area; two of these subpopulations were highly related. The microsatellite c1 subpopulation was more infective to *Ny. albimanus* and confined to coastal areas, while parasites exhibiting f1/f2 subpopulations predominately infected *An. pseudopunctipennis* on the foothills; the population structure followed that of the susceptible vector geographic distribution with limited gene flow (Joy et al., 2008). Furthermore, the genetic analysis of ookinete surface proteins Pvs25/Pvs28, involved in mosquito midgut invasion (Gonzalez-Ceron et al., 2010), revealed two main haplotypes that followed that of the principle populations identified using microsatellites. Parasites termed Types A and B corresponded to c1 and f1/f2 subpopulations, respectively, as they depicted similar geographical distributions and vector infectivity (Joy et al., 2008; Gonzalez-Ceron et al., 2010). Further studies using *Ny. albimanus* and *An. pseudopunctipennis* from other areas of Mexico and *An. pseudopunctipennis* from Guatemala indicated that the differential infectivity of the two southern Mexican *P. vivax* Pvs25 haplotypes (presumably types A and B) followed the same pattern, irrespective of the geographic origin of the mosquitoes (Gonzalez-Ceron et al., 2019).

An electron microscopy analysis of *P. vivax* (corresponding to those identified as PvSM-B) during ookinete migration in *Ny. albimanus* midguts revealed that a large portion of parasites were unable to invade and accumulated on the internal midgut surface. Other parasites within the midgut epithelium presented aberrant morphology and signs of damage. Those that reached the external midgut surface and initiated oocyst development interrupted their development and died. These observations suggest, in part, the participation of Pvs25/Pvs28 proteins and low levels of infectivity from Type B parasites (that resemble PvSM-B) in *Ny. albimanus* (Gonzalez-Ceron et al., 2001). Other ookinete proteins play essential roles in parasite development in the mosquito midgut (Dessens et al., 2003; Ramakrishnan et al., 2011). In this study, we observed a strong LD between gene polymorphisms, at codons Pvs25 130, Pvs28 87 and 110, SOAP 74, and CTRP 888 and 1754, and the ribosomal variant rV2. The polymorphisms in Pvs25 130 and Pvs28 87 confirmed our previous observations (Gonzalez-Ceron et al., 2010). Additionally, amino acid substitutions at CTRP between both lineages may be functionally relevant to the differential ookinete development in the two mosquito species. In this molecule, the change of hydrophobic Met at residue 888 to a basic polar Lys, may increase the positive charge in the von Willebrand Factor type A domain, critical for ookinete motility and oocyst formation (Ramakrishnan et al., 2011). At domain TPS1, residue 1754 Gly changed to Cys; a polar amino acid participating in disulfide bonding and essential to protein folding may confer a rearrangement in the protein structure.

Variation at SOAP, chitinase, and CelTOS is unlikely to contribute to the differential vector infectivity. The differential polymorphisms at SOAP in the lineages PvSM-A vs PvSM-B might support the differences in vector specificity; this protein interacts with the mosquito midgut laminin (Dessens et al., 2003), and the amino acid substitution did not seem to affect its molecular properties. Instead, it might reflect differences in the evolutionary history of these parasite lineages. In CelTOS, a mutation at codon Val118Lys found at a low frequency in PvSM-A was also reported in Brazil (Chaves et al., 2017) and Iran (Mehrizi et al., 2017), and it seems to be distributed globally (Hupalo et al., 2016). The amino acid substitutions Lys178Thr and Gly179Arg detected in southern Mexico have now been reported for the first time in Latin America, but they are common outside of the continent (Hupalo et al., 2016; Mehrizi et al., 2017). In fact, the divergent haplotype that expressed changes at codon 118, 178, and 179 was reported in Iran (Mehrizi et al., 2017).

The genetic and haplotype diversity suggests that PvSM-B was introduced more recently than PvSM-A in SM, and this was also inferred from previous results using microsatellites (Joy et al., 2008). Conversely, ongoing adaptations of this lineage were revealed by a subgroup (PvSM-Bb) that showed an increase in the proportion of infected *Ny. albimanus* along with a reduction in the infection rate and oocyst density in *An. pseudopunctipennis*. Although, the genetic markers analyzed here were not sensitive to discern this subgroup, further genomic analysis might provide new insights into this adaptation process. It is presumed that corresponding mosquito interacting molecules evolved separately during the approximately 100 million years that elapsed since the two genera diverted (Foster et al., 2017; Harbach, 2018). The polymorphisms in CTRP, Pvs25, and Pvs28 in PvSM-B suggest that, besides avoiding mosquito immunity, the parasite molecules might have been selected to better interact with their vectors during the critical steps to ensure infection. These signs of adaptive trade emphasize the plasticity of this parasite lineage, but their molecular mechanisms are in need of further clarification.

The high genetic differences between PvSM-B and PvSM-A, the clustering of PvSM-A with South American parasites, and the different infectivity patterns produced in the two main vectors in SM suggest that these lineages were under different vector selection and adaptation processes. The genetic similarities of PvSM-A with parasites from Peru and Colombia suggest a parasite flow between Mesoamerica and South America. This correlates with the low *F_ST_* value reported between Central and South American parasites using mitochondrial DNA (Taylor et al., 2013). The PvSM-A lineage in SM and *P. vivax* in Central America share the same infectivity to *Ny. albimanus*, (Warren et al., 1980; Li et al., 2001; Gonzalez-Ceron et al., 2010). Similarly, it was suggested that the *Nyssorhynchus* species conducted the *P. vivax* flow along the Northwest and South Pacific of Colombia (Pacheco et al., 2019). Accordingly, PvSM-A are predominant in Central and South America, probably due to the predominance of the *Nyssorhynchus* species in these regions; for example, *Ny. darlingi, Ny. aquasalis, Ny. nuñeztovari,* and *Ny. albitarsis* (Sinka et al., 2012; Conn et al., 2013; Fernández et al., 2014; Laporta et al., 2015; Ahumada et al., 2016) are all implicated as vectors of *P. vivax* (da Silva et al., 2006; Neves et al., 2013; Rios-Velasquez et al., 2013; Hurtado et al., 2018; Martins-Campos et al., 2018; Prussing et al., 2018).

Previous studies that analyzed the global population structure of *P. vivax* using whole genome parasite sequences, divided parasites into Old World and New World parasites. From the latter, a cluster comprised mostly of isolates from Mexico was purported to be of recent common ancestry (Hupalo et al., 2016). Also, a divergence signal between New World and Old World parasites was centered on the surface protein Pvs47 of gametes. The orthologue protein of *P. falciparum* participates in the evasion of mosquito immunity mediated by the complement-like thioester containing protein TEP1. A strong geographic structure in natural populations has been argued to be an indication of the parasite's adaptation to the new vector species (Molina-Cruz et al., 2017). Concurrently, it was proposed that the introduction of *P. vivax* to America during colonial times (Neafsey et al., 2012) involved adaptation to the local mosquito vectors, where, in South America, they are mainly composed of members of the *Nyssorhynchus* genus (Hupalo et al., 2016). A reduced haplotype diversity of Pvs47 in *P. vivax* samples from SA (Colombia, Peru, and Brazil) support this hypothesis. However, most (but not all) samples from SM were separated by the presence of two non-synonymous changes compared to other American parasites (Hupalo et al., 2016) and may denote a molecular adaptation to a different mosquito genus. Interestingly, we tracked three samples to belong to the PvSM-B lineage, which is more infective to *An. pseudopunctipennis* (**Supplementary Figure S6**). As expected, other isolates (apparently PvSM-A) from southern Mexico grouped with South American isolates (Hupalo et al., 2016).

On the other hand, PvSM-B may have come from a different genetic pool. A continuous trade between the Philippines and Mexico was initiated in 1565 and lasted for 250 years. The Nao of China, between Manila and Acapulco and on the Pacific coast of Mexico, carried out the exchange of goods and people (Guzmán-Rivas, 1960). This opens the possibility for the introduction of *P. vivax* from Asia or from trading routes comprising south Asia to an area were the genus *Anopheles* is abundant. An alternative route of introduction to North America might have been through slave trading.

Although the highly susceptible vector *An. pseudopunctipennis* is widely distributed from North America to Peru (Sinka et al., 2012), parasites closely related to PvSM-B lineage were not detected in South America. So far, we have documented the presence of PvSM-B only in southern Mexico, and, awaiting further sampling, we speculate that its distribution extends to other parts of North America and that vectors of the genus *Anopheles* transmit it. Although the USA eliminated malaria in 1940s and 50s, (Centers for Disease Control and Prevention [CDC], 2018), abundant *An. pseudopunctipennis* and *An. quadrimaculatus* coincide in Southeast-USA where most malaria cases were reported during late 1800s and early 1900s (Sinka et al., 2012). In these areas, different *Anopheles* species were present, e.g., *An. quadrimaculatus, An. freeborni, An. pseudopunctipennis*, and *An. punctipennis* (Young et al., 2008); the last two species are also found in north and/or south Mexico [Centers for Disease Control and Prevention (CDC), 2018]. *An. punctipennis* was found infected with *P. vivax* in southeast USA (Young et al., 2008). Through a couple of experiments, we documented the infectivity of *P. vivax* Pvs25 130Thr (likely PvSM-B lineage) to *An. punctipennis* captured in North Mexico, similar to that registered in *An. pseudopunctipennis* (unpublished data). Experimental infections also support the possibility of northern *P. vivax* adaptation to *Anopheles*. The St. Elizabeth strain, obtained in 1935 from a patient in South Carolina, USA (Collins, 2013), was highly infective to *An. quadrimaculatus* but non-infective to two strains of *Ny. albimanus* (from Florida and Panama) (Jeffery et al., 1954).

## Conclusions

High densities of *An. pseudopunctipennis* and *Ny. albimanus* mosquito species and two *Plasmodium vivax* lineages, PvSM-A and PvSM-B, which are defined by genes expressed in the ookinete stage, were present in southern Mexico. PvSM-A parasites were genetically similar to parasites in Central and South America and likely adapted and transmitted by *Nyssorhynchus* mosquito species. PvSM-B, meanwhile, was exclusive to southern Mexico, comprising parasites highly infective to *An. pseudopunctipennis.* This lineage comparts ancestry with parasites from Madagascar. This group probably represents an old lineage distributed further north of Mexico and USA and is more adapted to *Anophele*s species. The results suggest that these lineages evolved separately, likely by geographic and vector restriction. However, some PvSM-B parasites appear to present an adaptive trade-off to infect both vector species.

## Data Availability Statement

Gene sequences were deposited at NCBI Gen Bank database. All other relevant data are contained within the article or **Supplementary Material**.

## Ethics Statement

The protocol was approved by the Ethics Committee of the National Institute of Public Health, Mexico (CI1042). This study was carried out applying the bioethics guidelines (CITI program) and all participants above 18 years old gave their written informed consent, and the minors between 7-17 years old gave their assent accompanied by the written informed consent of one parent or the guardian, in accordance with the Declaration of Helsinki.

## Author Contributions

LG-C conceived and coordinated all aspects of the study and obtained funding. FS-V and MO-M performed the molecular analysis. CV-T performed the mosquito breeding. LG-C, JH-A, and JM-B implemented computational analyses (genetic and/or statistical). MCR provided comments and supervised the molecular analysis. LG-C and MHR interpreted data and prepared the manuscript with contributions from all co-authors. All authors approved the final version.

## Funding

This work was supported by CONACyT-Mexico: projects Salud-2004-01-119 and CB-2009-01-131247.

## Conflict of Interest

The authors declare that the research was conducted in the absence of any commercial or financial relationships that could be construed as a potential conflict of interest.

## Abbreviations

*P. vivax, Plasmodium vivax;* Sal I, Salvador I strain; *Ny. albimanus, Nyssorhynchus albimanus; An. pseudopunctipennis, Anopheles pseudopunctipennis;* CSP, Circumsporozoite protein; SOAP, Secreted ookinete adhesive protein; CTRP, Circumsporozoite-and thrombospondin-related adhesive protein; CelTOS, Cell-traversal protein for ookinetes and sporozoites; 18S rRNA, 18S small subunit ribosomal RNA type S; SM, southern Mexico; LD, linkage equilibrium; SNV, single nucleotide variation; ML, maximal likelihood.
