# Supplementary material for: Ookinete-Specific Genes and 18S SSU rRNA Evidenced in Plasmodium vivax Selection and Adaptation by Sympatric Vectors
Source: Front Genet. 2020 Feb 21;10:1362. doi: 10.3389/fgene.2019.01362 (PMC7047961; doi:10.3389/fgene.2019.01362)
Supplement: Supplementary file 1 [file Image_1.pdf]

A

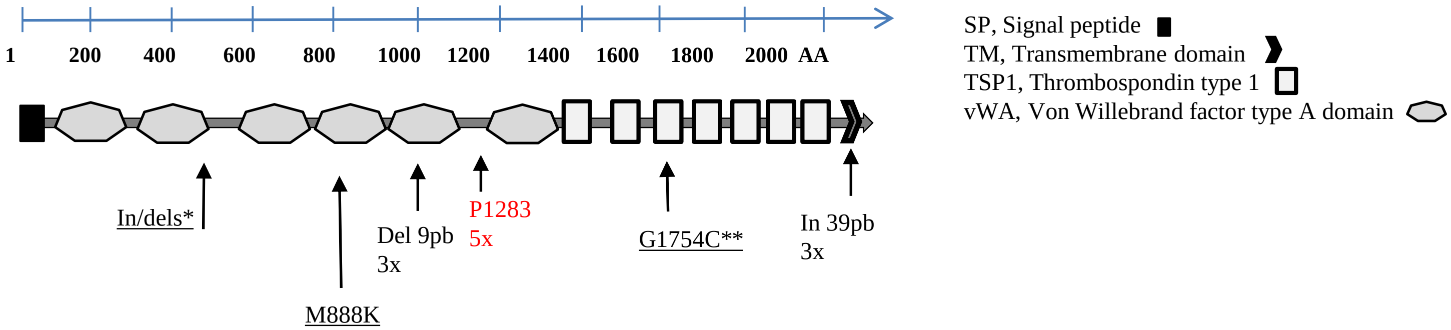

B

```
Codon:        535      In/dels*        585  1734                 1754**                     1781  2019
               |                        |    |                     |                          |     |
aXM001614511.1 KP------GGEEKPGS/PNGSDKPNE//KR---------------------------------------------AH//GG-------------SG
bAB247369.1    ..------......../.........//..VQRARLFSGGSSGGSGGSGGRGSGGAAHPPPSPAGSVPRPNPSSQ..//..-------------..
SM8101         ..GSSDKP......../..------.//..VQRARLFSGGSSGGSGGSGCRGSGGAAHPPPSPAGSVPRPNPSSQ..//..SGSGSGSGCSGSG..
cPvP01                                  // ..VQRARLFSGGS----------GSGGAAHPPPSPAGSVPRPNPSSQ..//
```

C

	In/dels* :	N isolates (codes):
Type 1	..------GGEEKPGS / PNGSDKPNE	1 isolate SM7401 (Sal I)
Type 2	..GSSDKP........ / ..------.	24 isolates (including SM8101)
Type 3	..------------.. /	2 isolates: SM6001, SM8203
Type 4	..------........ / ..------.	2 isolates: SM2804, SM15202

Supplementary Figure S1 *P. vivax* CTRP amino acid polymorphism in a southern Mexican isolate (SM). A) The Primary structure of the complete *ctrp* gene showing mutations detected in isolate SM8101 (compared to Sal I strain). **B)** Alignment of three reference sequences of Sal I strain (a,b), P01 strain (c) and isolate SM8101; a fragment of 135 bp at the carboxyl end was only present in reference AB247369 and all isolates from SM. **C)** Four different. In/Del* types were detected in SM samples. Dots indicate homology to the reference sequence; hyphens indicate absence of nucleotide or amino acid residue. AA, amino acid.
