# Supplementary material for: Ookinete-Specific Genes and 18S SSU rRNA Evidenced in Plasmodium vivax Selection and Adaptation by Sympatric Vectors
Source: Front Genet. 2020 Feb 21;10:1362. doi: 10.3389/fgene.2019.01362 (PMC7047961; doi:10.3389/fgene.2019.01362)
Supplement: Supplementary file 2 [file Image_2.pdf]

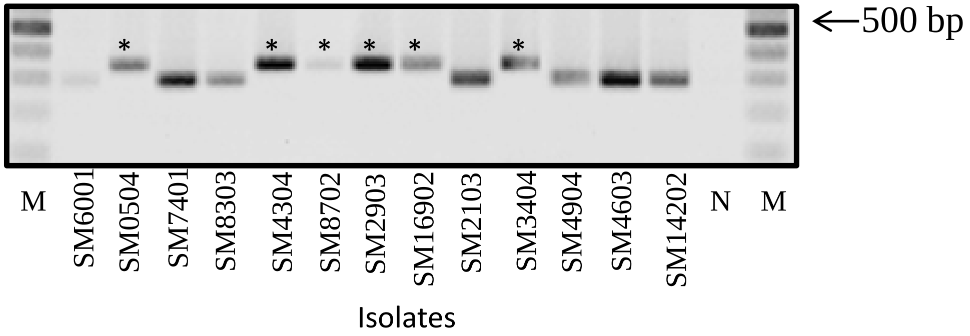

Supplementary Figure S2 Gel image of chitinase gene fragment variation. The image shows two amplified products of ≈ 300 bp (Chit 1) and ≈ 354bp* (Chit 2). N, control negative. M, molecular marker 100 bp.
