# Supplementary material for: Ookinete-Specific Genes and 18S SSU rRNA Evidenced in Plasmodium vivax Selection and Adaptation by Sympatric Vectors
Source: Front Genet. 2020 Feb 21;10:1362. doi: 10.3389/fgene.2019.01362 (PMC7047961; doi:10.3389/fgene.2019.01362)
Supplement: Supplementary file 3 [file Image_3.pdf]

A

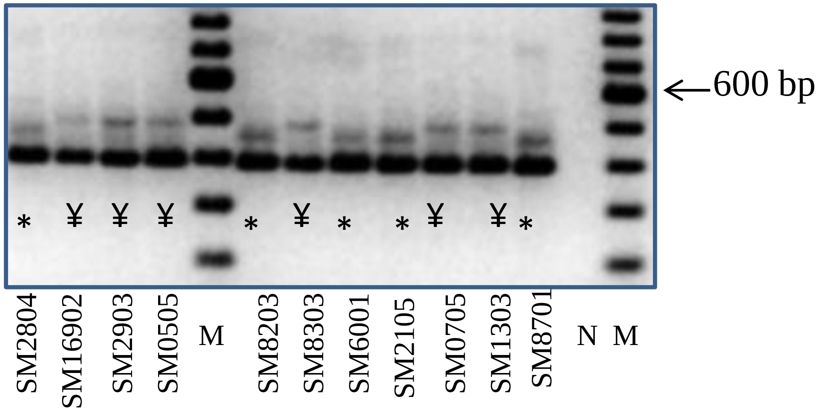

B

```
Isolate:     5′ →1790                                                                                  1852 ← 3′
Sal-I_rV2      ATATGTACAA CGAGTTATTA AAATTACG-- ---------- --------AT TCAGCTT--- ----GCTGTT ACGTATTTTT CCTCCACTGA
SM8701**       .......... .......... ........-- ---------- --------.. .......--- ----...... .......... ..........

Thai_rV1       .......... .......C.. G..A..T.CA TTTCGCTATT TTGTGCTT.. C....C.TTT TAGCAT.A.G G.A..C.... ..........
SM5703         .......... .......C.. G..A..T.CA TTTCGCTATT TTGTGCTT.. C....C.TTT TAGCAT.A.G G.A..C.... ..........
SM8303         .......... .......C.. G..A..T.CA TTTCGCTGTT TTGTGCTTT. C....C.TTT TAGCAT.A.G G.A..C.... ..........
             5′ →1650                                                                                  1739 ← 3′
```

Supplementary Figure S3 *P. vivax 18S* rRNA S-type size variation.

A. Gel image of ribosomal S-type gene fragment size variation. N, negative control. M, molecular marker 1 Kb plus. ¥ rV1 and *rV2.

B. *P. vivax* 18S rRNA type-S gene sequence alignment of isolates variants rV2 (Sal I) or rV1 (Thai). Variable fragment nt 1790-1852 is shown. B. Sequence size variation of rV1 versus rV2 sequences. **Other sequences were similar (PvSM-A: SM2804, SM2105, SM8203, SM15202).
