# Supplementary material for: Ookinete-Specific Genes and 18S SSU rRNA Evidenced in Plasmodium vivax Selection and Adaptation by Sympatric Vectors
Source: Front Genet. 2020 Feb 21;10:1362. doi: 10.3389/fgene.2019.01362 (PMC7047961; doi:10.3389/fgene.2019.01362)
Supplement: Supplementary file 4 [file Image_4.pdf]

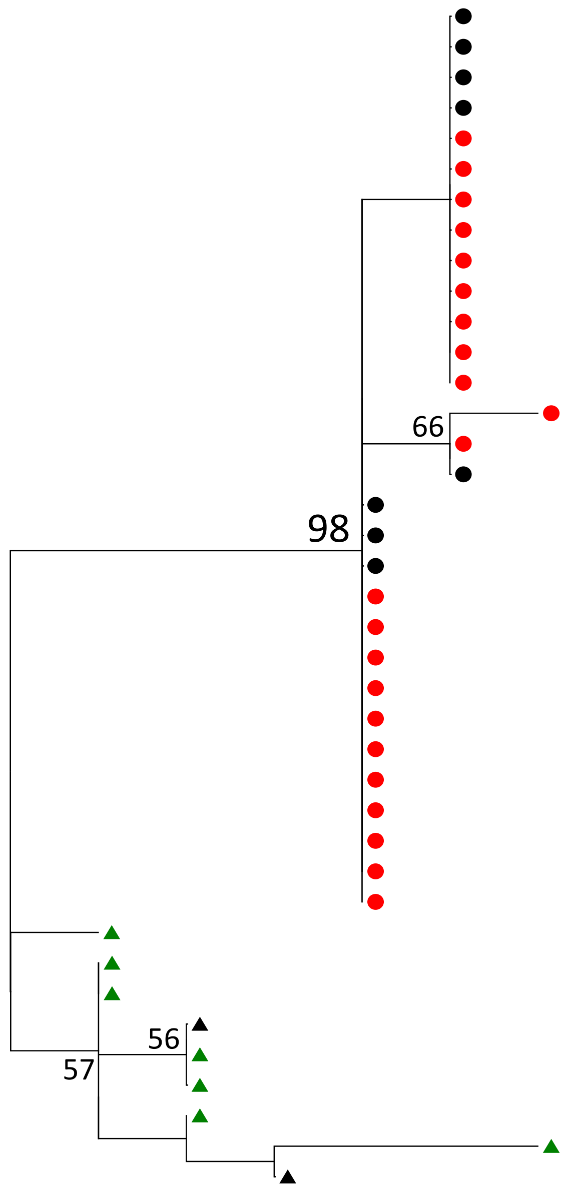

Supplementary Figure S4 . Maximal likelyhood phylogenetic tree contructed with concatenated sequences of *P. vivax* ookinete specific-genes from southern Mexico. Twenty nine sequences comprising 2478bp from this study plus 10 sequences obtained from PlasmoDB (in black); this ML tree did not comprise SNP at codon 1754 of CTRP. It shows two main branches supported by 98% bootstraping similar to Figure 1. Bootstrapping above 50% are shown.
