# Supplementary material for: Ookinete-Specific Genes and 18S SSU rRNA Evidenced in Plasmodium vivax Selection and Adaptation by Sympatric Vectors
Source: Front Genet. 2020 Feb 21;10:1362. doi: 10.3389/fgene.2019.01362 (PMC7047961; doi:10.3389/fgene.2019.01362)
Supplement: Supplementary file 5 [file Image_5.pdf]

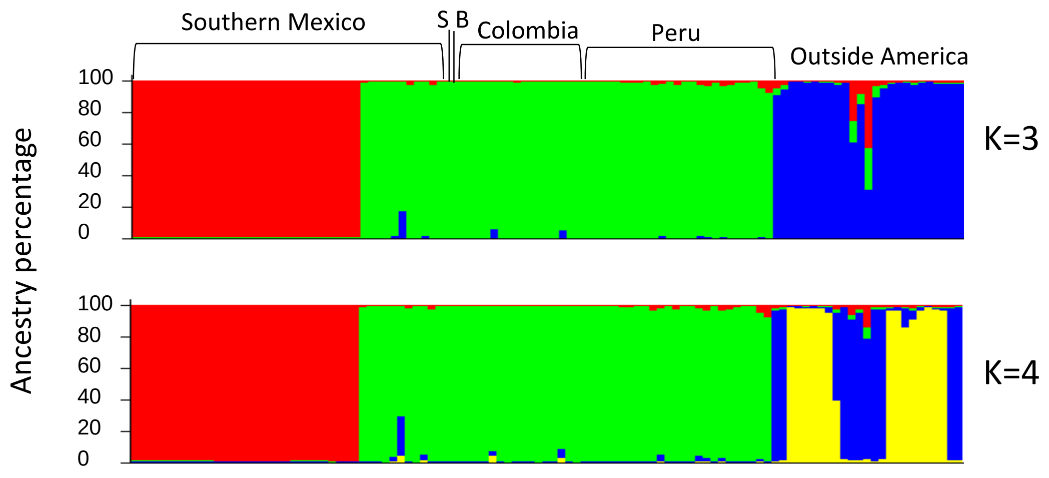

K	Reps	Mean LnP(K)	Stdev LnP(K)	Ln'(K)	|Ln''(K)|	Delta K
2	20	-766.385000	27.074989	—	—	—
3	20	-553.770000	0.447331	212.615000	180.92500	404.454178
4	20	-522.080000	3.626017	31.690000	35.340000	9.746231
5	20	-525.730000	12.172579	-3.650000	17.420000	1.431085
6	20	-546.800000	35.360445	-21.070000	—	—

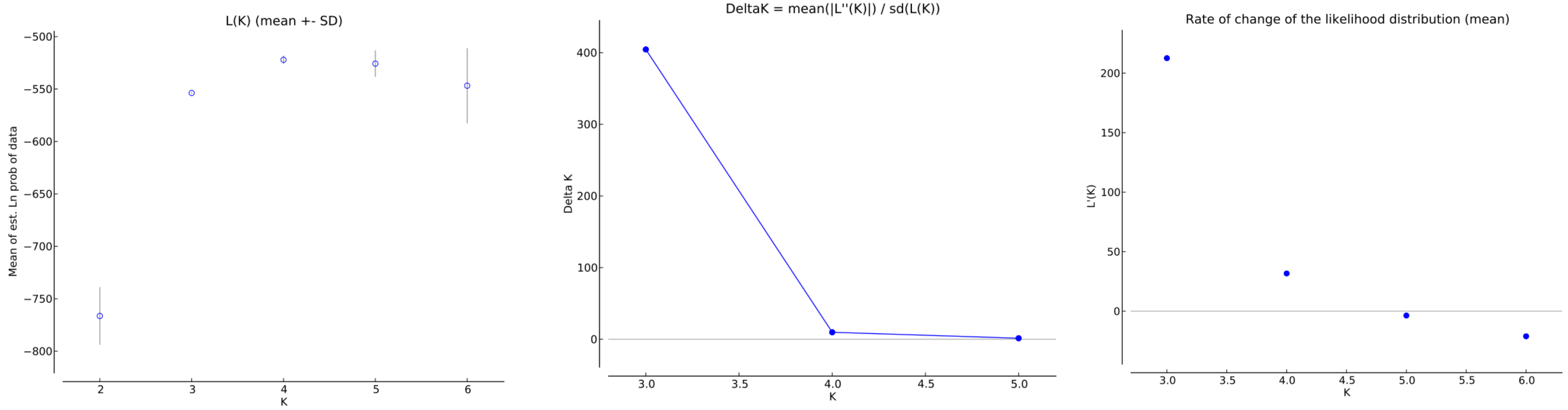

Supplementary Figure S5 Genetic structure analysis of global concatenated gene sequences of *P. vivax* ookinete specific-genes. Comparing results from K=3 and K=4. It shows that American parasites clustered into two populations; 109 worldwide concatenated sequences of 2478 bp were included.
