# Supplementary material for: Ookinete-Specific Genes and 18S SSU rRNA Evidenced in Plasmodium vivax Selection and Adaptation by Sympatric Vectors
Source: Front Genet. 2020 Feb 21;10:1362. doi: 10.3389/fgene.2019.01362 (PMC7047961; doi:10.3389/fgene.2019.01362)

Supplementary Figure S6 Phylogenetic tree of *Pvs47* gene sequences of *Plasmodium vivax* worldwide isolates. Maximum Likelihood method based on the HKY+G model. The phylogenetic reconstruction tree was run with 1000 replicates.

A branch of 67% bootstraps separates all isolates of America from other geographic sites, and another branch of 64% bootstraps separated a set of 12 Mexican isolates including three belonging to lineage PvSM-B (**). One Mexican isolate was traced back to lineage PvSM-A (*) that clustered with American isolates. This resembled the clustering of Figure 2.

The analysis involved 100 nucleotide sequences of the complete gene 1302 bp in the final dataset. Data source: https://plasmodb.org/.

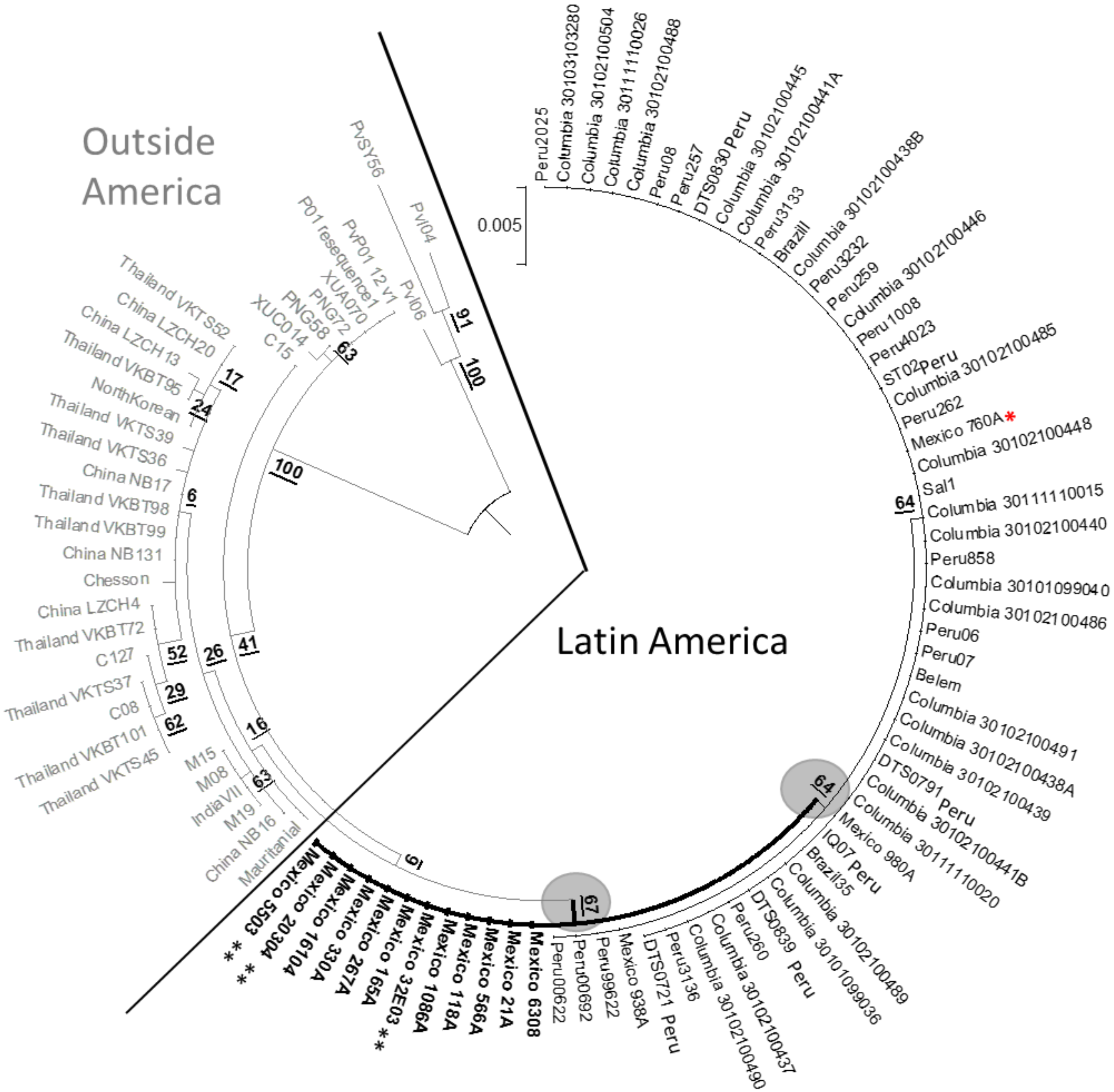

Supplement: Supplementary file 6 [file Image_6.pdf]
