# Supplementary material for: Ookinete-Specific Genes and 18S SSU rRNA Evidenced in Plasmodium vivax Selection and Adaptation by Sympatric Vectors
Source: Front Genet. 2020 Feb 21;10:1362. doi: 10.3389/fgene.2019.01362 (PMC7047961; doi:10.3389/fgene.2019.01362)
Supplement: Supplementary file 7 [file Table_1.pdf]

Supplementary Table S1 Primers designed and used to amplify genes of proteins expressed at the *P. vivax* ookinete stage

Accession number (Gen Bank)	Primer name	Oligonucleotide
pvs25; Chr 6	Pvs25-F23	5´ GTG TAT GTG TAA CGA AGG GCT 3´
(XM_001608410.1)	Pvs25-R214	CAG TTT CTC CCG TTT TGG TA
Pvsoap; Chr 13	SOAP-F	AGA AGC TTA AGC TCT GAC ATA ACC TCA
(XM_001616857)	SOAP-R	TGA ATT CTT ACA ATA ACA GGA GCA GCT G
Pvchitinase; Chr 1	PvChit-F1	TTC GCC AAC CTG TCT GCC TT
(XM_001613347)	PvChit-R1	CCC ATT TGT TGA GAC AAG TAG TTC G
	PvChit-F2	CGG AGT GAT GCT AAA CGA AAT CAG
	PvChit-R2	CTG CAC ACC TGC TCG CTT CTC
	Rep-Chit-F	GGC TTA TAC ACG GAC GAG TCC A
	Rep-Chit-R	CTC TTG GAG GTT TTC TAT CAT GGC TA
Pvctrp; Chr 8	CTRP-4100-F	CAG GAT CCA CAG CGT AGC CAA GAT G
(AB47369.1)	CTRP-4099-R	GAA CTC GAG ACT GCC GCT ACC ACT T
	CT-1F	CGA CGA ATC TGG AAG CAT AGG AAT
	CT-1R	CAG GGC TTG CAC AAT GAA GGT T
	CT-2F	GCGTTTCGATGGCTGATGAGA
	CT-2R	CACCATCACATACGTTCCTGCCTA
	CT-3F	TAG GCA GGA ACG TAT GTG ATG GT
	CT-3R	CTC CCC AGT CTT CCC ACT CGT
	CT-4F	TCC GGA AGC ATT ACC CTG AAC AAG
	CT-4R	TCC CAG TCG CAC TTG ATG ACC
	CT1754-F nt 5040-5063	ATA CTC CAC GAG AAG CAG GAC GTT
	CT1754-R nt 5472-5495	ATC CTC ATT CCG ACG TTG CAC TTG
	CT18rF nt1520-1538	ACC AGC TCA CCA GCA TAA C
	CT18rR nt2077-2098	ATA CCA GGG CTT GCA CAA TGA
