# Supplementary material for: Ookinete-Specific Genes and 18S SSU rRNA Evidenced in Plasmodium vivax Selection and Adaptation by Sympatric Vectors
Source: Front Genet. 2020 Feb 21;10:1362. doi: 10.3389/fgene.2019.01362 (PMC7047961; doi:10.3389/fgene.2019.01362)
Supplement: Supplementary file 9 [file Table_3.pdf]

Supplementary Table S3 Gene sequences extracted from PlasmoDB database and used in this study.

Location	Country	year of collection	sample code
Latin America	BRAZIL	1980	Belem
	Colombia	2012	Columbia_30101099036
		2012	Columbia_30101099040
		2012	Columbia_30102100437
		2012	Columbia_30102100439
		2012	Columbia_30102100440
		2012	Columbia_30102100445
		2012	Columbia_30102100446
		2012	Columbia_30102100448
		2013	Columbia_30102100485
		2013	Columbia_30102100486
		2013	Columbia_30102100488
		2013	Columbia_30102100489
		2013	Columbia_30102100490
		2013	Columbia_30102100491
		2013	Columbia_30102100504
		2013	Columbia_30103103280
		2013	Columbia_30111110015
		2013	Columbia_30111110020
		2013	Columbia_30111110026
	Southern Mexico	2002	Mexico_118-A
		2004	Mexico_161-04
		2002	Mexico_165-A
		2002	Mexico_21-A
		2002	Mexico_267-A
		2002	Mexico_330-A
		2002	Mexico_566-A
		2008	Mexico_63-08
		2000	Mexico_938-A
		2001	Mexico_980-A
	Peru	2011	Peru00622
		2011	Peru00692
		2011	Peru00699
		2008	Peru06
		2008	Peru07
		2008	Peru08
		2010	Peru1008
		2010	Peru2025
		2013	Peru257
		2013	Peru259
		2013	Peru260
		2013	Peru262
		2011	Peru3043_D0
		2011	Peru3133

		2011	Peru3136
		2012	Peru3232
		2012	Peru3270_D43
		2012	Peru4023
		2008	Peru858
		2008	Peru872
		?	DTS0721
		?	DTS0791
		?	DTS0830
		2012	DTS0839
Outside America	Cambodia	2011	C127
	China	?	China_LZCH-13
		2013	China_LZCH-20
		2013	China_LZCH-4
		2012	China_NB-17
	Madagascar	2010	M08
		2010	M15
		2010	M19
	North Korea	1953	NorthKorean
	PapuaNew Guinea	1944	Chesson
		2009	PNG58
		2010	PNG72
		2012	XUA070
		2012	XUC014
	Thailand	2013	Thailand_VKBT-100
		2013	Thailand_VKBT-101
		2012	Thailand_VKBT-72
		2012	Thailand_VKBT-95
		2013	Thailand_VKBT-98
		2013	Thailand_VKBT-99
		2012	Thailand_VKTS-36
		2012	Thailand_VKTS-37
		2012	Thailand_VKTS-39
		2013	Thailand_VKTS-52
	Indonesia	2011	P01_resequence1
